# Physical activity coaching programme for people with Long COVID: a pilot randomised clinical trial

**DOI:** 10.1038/s41598-026-44806-9

**Published:** 2026-03-24

**Authors:** Nicola S. Diciolla, Alda Marques, Ana Jiménez-Martín, Evelyn Alecto-Aznar, María Torres-Lacomba, María José Yuste-Sánchez

**Affiliations:** 1 Physiotherapy in Women’s Health Research Group – FPSM, Department of Nursing and Physiotherapy, University of Alcalá, Campus Científico-Tecnológico, Crta. de Madrid-Barcelona, Km. 33, 600, Alcalá de Henares, Madrid, 28805 Spain; 2 Respiratory Research and Rehabilitation Laboratory – Lab3R, School of Health Sciences – ESSUA, University of Aveiro, Campus Universitário de Santiago, Agras do Crasto, Building 30, Aveiro, 3810-193 Portugal; 3 Institute of Biomedicine – iBiMED, University of Aveiro, Campus Universitário de Santiago, Agras do Crasto, Building 30, Aveiro, 3810-193 Portugal; 4 Electronics Engineering applied to Smart Spaces and Intelligent Transportation Systems Research Group - GEINTRA, Department of Electronics, University of Alcalá, Campus Científico-Tecnológico, Crta. de Madrid-Barcelona, Km. 33, 600, Alcalá de Henares, Madrid, 28805 Spain; 5 Ramón y Cajal Institute of Health Research – IRYCIS, Hospital Universitario Ramón y Cajal, Ctra. Colmenar Viejo, Fuencarral-El Pardo, Madrid, 28034 Spain

**Keywords:** Long COVID, Physical activity, Feasibility trial, Behavioural intervention, Remote coaching, Randomised controlled trial, Diseases, Health care, Medical research, Signs and symptoms

## Abstract

Low physical activity-PA may affect health outcomes in people with Long COVID. This study assessed the feasibility, safety and preliminary efficacy and effectiveness of a PA coaching programme vs. usual care. A randomised, parallel-group, pilot trial compared a twelve-week multicomponent PA coaching intervention (EG: self-monitoring, feedback, and goal setting/review) with usual care (CG: self-managed PA). PA (inertial sensors-light/moderate/vigorous PA [min·day^-1^], steps·day^-1^), functional capacity (6-minute walk test-6MWD; 1-minute sit-to-stand test-1minSTS), dyspnoea (modified Medical Research Council scale-mMRC), fatigue (Functional Assessment of Chronic Illness Therapy-Fatigue scale-FACIT-FS) and health-related quality of life-HRQoL (European Quality of Life-Five Dimensions-Five Levels visual analogue scale-EQ-5D-5L) were assessed at baseline, three- and six-months afterwards. Recruitment (≥70%), retention, adherence (≥80%) and preliminary efficacy/effectiveness explored feasibility. Adverse events determined safety. Fifty participants (EG: n=25, 47±10y, 80%♀; CG: n=25, 46±11y, 76%♀) were enrolled. Recruitment (89%), retention (92%) and adherence (83%) met feasibility criteria. At three-months, the EG improved PA, sedentary behaviour and functional capacity compared to the CG (light PA: +53[19;89]min·day^-1^; steps·day^-1^: +4632[2626;6638]; sedentary time: -71[-141;-2]min·day^-1^; 6MWD: +119[51;187]m). Dyspnoea, fatigue and HRQoL also improved (mMRC: -1[-2;-1]; FACIT-FS: +11[6;17]; EQ-5D-5L: +21[13;29]). These changes were maintained or further improved at six-months/three-months post-intervention (light PA: +73[36;111]min·day^-1^; moderate-to-vigorous PA: +7[1;14]min·day^-1^; steps·day^-1^: +5236[3070;7402]; sedentary time: -98[-165;-31]min·day^-1^; 6MWD: +136[69;203]m; 1minSTS: +9[2;15]reps; mMRC: -1[-2;-1]; FACIT-FS: +19[13;24]; EQ-5D-5L: +25[18;33]). No adverse events occurred. The PA coaching programme was feasible, safe and may enhance and sustain PA, sedentary behaviour, functional capacity, symptoms, and HRQoL in Long COVID up to three-months post-intervention. A large trial is warranted. ClinicalTrials.gov: NCT06165978

**Supplementary Information:**

The online version contains supplementary material available at 10.1038/s41598-026-44806-9.

## Introduction

Long COVID is a multisystem disorder affecting a significant proportion of people post-SARS-CoV-2 infection, with persistent symptoms such as fatigue, dyspnoea and cognitive dysfunction^1–3^. These symptoms, often cyclical and triggered by physical or mental exertion, may prevent engaging in daily activities^4,5^. Indeed, more symptomatic people tend to be less physically active (individuals with four-symptoms vs. those with one/no-symptoms: 6464±2665 vs. 12290±5872 steps·day^-1^), reduce physical activity-PA and engage in sedentary behaviour, to adjust to lower PA levels^6,7^, leading to progressive physical inactivity, sedentary behaviour, worsened symptoms, increased morbidity and healthcare burden^8,9^.

Physical inactivity is a well-recognised predictor of functional decline, disability, morbidity and premature mortality, contributing to up to sixteen million deaths worldwide^10^. In chronic respiratory diseases and Long COVID, symptoms of dyspnoea and fatigue often trigger activity avoidance and physical deconditioning, strengthening a self-perpetuating downward spiral that exacerbates symptoms and disease progression^2,4,11–17^. This cycle increases the risk of exacerbations, hospitalisation and mortality, highlighting the need for interventions that support safe and sustainable PA engagement.

Pulmonary rehabilitation and exercise-based interventions, whether delivered face-to-face or remotely, have shown potential benefits for functional capacity, peripheral muscle strength, dyspnoea, fatigue and health-related quality of life-HRQoL in sub-acute and Long COVID^18–21^. However, existing studies have largely focused on short-term outcomes, often neglecting post-exertional symptom exacerbations^21–23^. These exacerbations raise significant safety concerns and may discourage participation, prompting poor adherence and dropout rates of up to 50%^22,24^. Furthermore, a recent report (n=100, 51±11 years, 73% female, 13±5 months post-COVID-19) found no significant changes in PA and sedentary behaviour following a three-week inpatient rehabilitation programme^25^. Therefore, strategies specifically targeting PA behaviour are much needed. PA coaching interventions have shown efficacy in promoting sustainable PA engagement and self-management in chronic respiratory diseases^26–28^. These approaches are grounded in established theoretical frameworks, e.g., the behaviour change wheel^29^, which conceptualise behaviour as a function of capability, opportunity and motivation, and employ behaviour-change techniques-BCTs (e.g., self-monitoring, personalised feedback, collaborative goal setting, action planning, graded progression)^30–32^. By addressing these factors, coaching interventions actively and safely support individuals in adopting and maintaining a physically active behaviour and therefore may offer a promising alternative for people with Long COVID. Nevertheless, their feasibility, acceptability and potential effects remain unexplored in this population.

Thus, this study aimed to assess the feasibility, and explore the preliminary efficacy and effectiveness, of a PA coaching programme vs. usual care on PA, sedentary behaviour and related health outcomes in people with Long COVID.

## Methods

### Study design

A pilot randomised controlled trial, single-blind (assessor-masked), was conducted, and adhered to the Consolidated Standards of Reporting Trials-CONSORT^33,34^ and Template for Intervention Description and Replication-TIDieR^35^ guidelines. All participants provided written informed consent, and data protection followed European regulations, ensuring anonymity through file encoding. Ethical approval was granted by the Ethics Committee for Research and Animal Experimentation, University of Alcalá (CEID/2023/3/072), and the study was registered on *clinicaltrials.gov* (NCT06165978; 12/12/2023). All methods were performed in accordance with the relevant guidelines and regulations, including the Declaration of Helsinki.

### Participants

Participants were recruited from the *Asociación Madrileña de COVID Persistente-AMACOP*, a Long COVID patient advocacy group in Madrid, Spain, between April and September 2024. Recruitment was conducted through the association newsletters and social media, with study materials co-designed with patient representatives. Inclusion criteria included adults (≥18 years old) with a confirmed COVID-19 diagnosis via polymerase chain reaction or protein N test, and a post-COVID-19 condition/Long COVID diagnosis, defined by reporting persistent symptoms for at least two-months following laboratory-confirmed, probable or suspected COVID-19 infection, lasting for at least three-months post-infection^1,36^. Diagnosis was confirmed by an experienced respiratory physiotherapist. Exclusion criteria included participation in another trial addressing PA or sedentary behaviour in the previous six-months, or severe cognitive, cardiovascular, neurological, or musculoskeletal conditions limiting participation.

### Interventions

#### Physical activity coaching programme

The experimental group-EG received a twelve-week PA behavioural intervention, informed by a systematic review with meta-analysis^28^, based on established behaviour-change frameworks (i.e., the behaviour change wheel)^29^ and recognised BCTs^30–32^, and co-designed with the research department of the patient advocacy group to address patient specific needs.

**Fig. 1:**
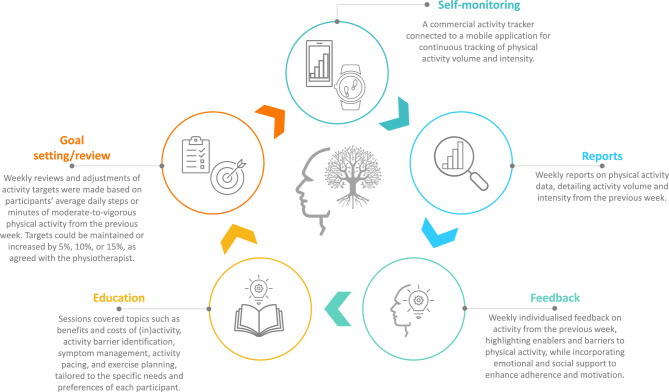
Description of the physical activity coaching programme for people with Long COVID. COVID: Coronavirus Disease.

Participants received one-to-one coaching session, once weekly, delivered remotely (video or telephone), with each session lasting approximately 30 minutes. The programme consisted of (Fig. 1):

- Self-monitoring (BCT: self-monitoring of behaviour; control theory): Participants were provided with an activity tracker (*Xiaomi Smart Band 8, Xiaomi Corporation, Beijing, China*) connected to a mobile application (*Mi Fitness, Xiaomi Corporation, Beijing, China*) for continuous monitoring of activity volume (steps·day^-1^ and time spent in moderate-to-vigorous PA [min·day^-1^]) and intensity (heart rate, walking speed and cadence). The application enabled goal setting, real-time feedback (alerts for goal achievement, or prolonged sedentary time), and daily, weekly and monthly PA data access.
- Reports (BCT: feedback on behaviour; control theory): Weekly PA data were extracted from the application by the end of the week and reviewed by the intervention provider, to monitor activity progression and inform session contents.
- Feedback (BCT: feedback on behaviour; control theory, social-support theory, motivational interviewing): Individualised weekly feedback based on PA reports was provided during the session, at the beginning of the week, with a focus on reinforcing positive behaviours, and supporting motivation and individual capability.
- Education (BCT: information about health consequences; information-motivation-behavioural model, social-cognitive theory, operant conditioning): Educational contents were tailored to individual needs, in line with current guidelines ^37^. Topics included symptoms recognition and management, PA and sedentary behaviour (enablers/barriers), pacing and graded activity strategies, and safe exercise planning. Education was delivered during each session, and was adapted to participants’ health literacy and educational level, and supplemented with written materials provided via text messages or email.
- Goal setting/review (BCT: goal setting, action planning; control theory, operant conditioning): At the end of each session, weekly PA targets were collaboratively reviewed, agreed and adjusted (+0%, 5%, 10%, or 15%) based on participants’ average PA levels (steps·day^-1^ and time spent in moderate-to vigorous PA [min·day^-1^]) and symptom burden during the previous week.

The intervention was delivered by registered physiotherapists specialised in cardiac and respiratory rehabilitation, and experienced in the management of chronic respiratory disease and Long COVID. Before study initiation, intervention providers completed standardised training, including familiarisation with intervention manual, BCTs, symptom-responsive pacing principles and safety procedures.

Intervention fidelity was supported using a structured intervention protocol and standardised session templates, with flexibility limited to individual goal setting and symptom-responsive graded activity. Session completion and content delivery were documented using standardised records, and any modifications were recorded with justification, timing and implementation details.

#### Usual care

The control group-CG followed a twelve-week self-management programme, autonomously setting their own activity targets. Participants were informed of and encouraged to align with the World Health Organization PA recommendations (i.e., 150–300min·wk^-1^ of moderate-to-vigorous PA)^38^. To ensure equivalent measurement conditions, participants received the same activity tracker and submitted weekly PA reports. No additional behavioural contents (e.g., individual sessions, feedback, support or education) was provided.

All participants were asked to report access to healthcare services (e.g., medical consultations, emergency visits, hospitalisations) and any adverse events, whether considered related to the intervention or not, to ensure safety monitoring. Intervention adherence was assessed through PA reports, physiotherapist feedback records, and participant compliance logs.

### Randomisation, allocation concealment and blinding

Participants were randomly allocated to either the PA coaching programme or usual care. The randomisation sequence was generated using block randomisation with 1:1 allocation ratio and variable block sizes, allowing between one and four participants per group within each block. The sequence was generated using R statistical software (*R: v.4.4.2, R Foundation, Vienna, Austria; RStudio: v.2024.12.0+467, PBC, Boston, MA, USA*). An independent researcher, not involved in participant recruitment, outcome assessment or intervention delivery, generated and safeguarded the allocation sequence. Allocation concealment was ensured using sequentially numbered, opaque, sealed envelopes. Outcome assessors were blinded to group allocation, although participants and intervention providers were not, due to the nature of the intervention.

### Outcomes

**Fig. 2:**
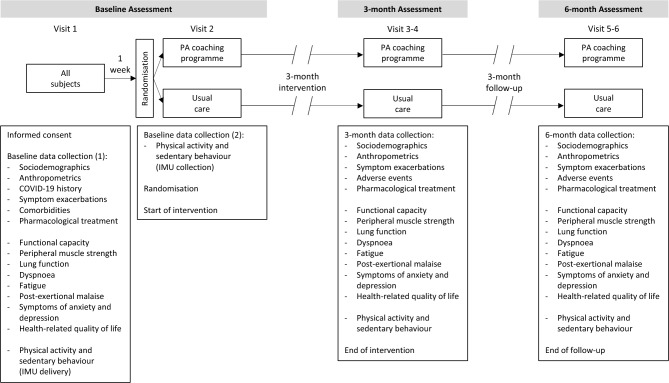
Study visits and assessments of the physical activity coaching programme for people with Long COVID. COVID-19: Coronavirus Disease 2019; IMU: inertial measurement unit.

Assessments were conducted at baseline, three- and six-months afterwards (Fig. 2).

### Primary outcome: feasibility

Recruitment was assessed as the rate of enrolled participants vs. eligible participants approached. Retention (acceptability) was evaluated as the rate of study completers vs. participants enrolled. Adherence (participant engagement) was measured through session attendance (sessions attended vs. planned) and activity completion (weekly targets achieved vs. intervention weeks). Adverse events (reinfections, symptom exacerbations, emergency visits, hospitalisations) were recorded.

### Secondary outcomes

#### Physical activity and sedentary behaviour

PA and sedentary behaviour were assessed using a custom-designed inertial measurement unit (triaxial accelerometers and gyroscopes), worn on the right hip during waking hours (e.g., 7.00am-10.00pm) over seven consecutive days. A valid assessment required a minimum of eight-hours·day^-1^ on at least four-weekdays^39^. Participants received verbal and written instructions and logged wear time interruptions. Wear-time compliance and data quality were monitored throughout the assessment period. Logged wear-time was cross-checked with device-derived non-wear indicators (e.g., prolonged zero-activity intervals and gyroscope inactivity) to ensure that all included days met predefined validity criteria.

Data were processed using MATLAB (*MathWorks. MATLAB (R2024a). Natick (MA): The MathWorks, Inc.; 2024*). PA levels were classified using a semi-automated, supervised machine learning approach. A random forest classifier, trained on labelled data from 33 healthy volunteers across 77 sessions of controlled activity (e.g., lying, sitting, walking, running), achieved 97% classification accuracy using subject-split validation. Step counts were derived from a tailored peak-detection algorithm applied to linear acceleration signals, with thresholds optimised for activity level (light, moderate, vigorous). Step detection parameters included minimum peak height, inter-step interval and signal prominence. The same peak-detection thresholds were applied to all participants, with no tailored adjustment for gait characteristics or walking cadence. The step-counting algorithm was validated against manual counts in 11 healthy volunteers across five walking and running trials, yielding a mean absolute percentage error of 3.5%. These validation procedures were conducted under both laboratory and overground conditions. Validation was not performed in people with Long COVID, and this may have affected the accuracy of peak-detection algorithm trained on healthy volunteers, because of potential gait impairments in this population (e.g., lower cadence, shuffling). However, similar inertial systems have shown validity and reliability in other clinical populations with potentially reduced or altered activity patterns (e.g., COPD)^40^. From the processed data, average daily time spent in light and moderate-to-vigorous PA, step count, and sedentary time (adjusted for daylight hours) were extracted. The proportion of participants walking fewer than 5000steps·day^-1^ was also computed^41^.

#### Functional capacity, peripheral muscle strength, symptoms and health-related quality of life

Functional capacity was evaluated using the six-minute walking test/distance-6MWT/6MWD^42^ and the one-minute sit-to-stand test-1minSTS^43^, as recommended. Peripheral muscle strength was assessed with the quadriceps maximal voluntary contraction-QMVC using a hand-held dynamometer (*MicroFET2, Acme Corporation, Springfield, USA*)^44,45^ and with the handgrip using a digital dynamometer (*EG101, Camry instruments, China*)^46^, as recommended. Maximal respiratory pressures-PImax and PEmax were assessed with a digital manometer (*MicroRPM®, Vyaire Medical GmbH, Hoechberg, Germany*), following current guidelines^47^.

Percentages predicted^48–52^ and proportions of people with impaired functional capacity and peripheral muscle strength (values ≤70pp) were computed.

Dyspnoea, fatigue, post-exertional malaise, anxiety and depression were measured using the modified Medical Research Council-mMRC dyspnoea scale^53^, the Functional Assessment of Chronic Illness Therapy-Fatigue-FACIT-FS^54^, the DePaul Symptom Questionnaire-DSQ^55^ and the Hospital Anxiety and Depression Scale-HADS^56^, respectively. HRQoL was explored using the European Quality of Life-Five Dimensions-Five Levels-EQ-5D-5L questionnaire^57^.

Proportions of participants with clinically relevant symptoms (mMRC≥2, FACIT-FS≤43, DSQ-criteria for post-exertional malaise and myalgic encephalomyelitis/chronic fatigue syndrome, HADS≥8^53–56^) and impaired HRQoL (EQ-5D-5L visual analogue scale ≤74 for women, ≤78 for men^58^) were determined.

Sociodemographics (age, sex) and anthropometrics (body mass index) were recorded. COVID-19-history (time since acute COVID-19, exacerbations), comorbidities (classified using the International Classification of Disease, 11^th^ Ed., and Charlson Comorbidity Index^59,60^), and pharmacological treatment (categorised using the Anatomic, Therapeutic and Chemical classification^61^) were also registered. Lung function (forced expiratory volume in one second and force vital capacity, as percentage predicted-FEV_1_pp and FVCpp) was assessed via spirometry, following the European Respiratory Society/American Thoracic Society guidelines ^62^).

### Feasibility criteria

Progression to a full trial required: ≥70% recruitment rate, ≥80% retention and adherence rates (≥80% of planned sessions and meeting weekly activity targets)^63^, no significant between-group differences in adverse events, and acceptable variability in secondary outcomes.

### Statistical analyses

Sample size was estimated using G*Power v3.1.9.4 (Universität Kiel, Germany, 2019) to achieve a reasonable precision level around the expected effect size derived from previous PA interventions in similar populations (mean difference ≈ 805±2144 or 807±1171 steps·day⁻1; Cohen’s f = 0.39)^28,64^. Forty-eight participants (24/group) were estimated as a pragmatic compromise between feasibility and estimation precision, allowing for a 20% attrition rate based on previous trials in people with myalgic encephalomyelitis/chronic fatigue syndrome and Long COVID^65–67^. Although repeated-measures ANCOVA analyses (α=0.20; 80% power) guided sample size computation, this was not intended for hypothesis testing but rather to ensure adequate precision in estimating variability. The α=0.20 is consistent with early-phase and pilot studies guidelines, allowing greater flexibility in balancing Type I and Type II errors in small exploratory samples^68–70^. In line with pilot trial methodology^33,71,72^, sample size was therefore driven by feasibility objectives and parameter estimation rather than hypothesis testing. For continuous outcomes, 15–25 participants/group are considered sufficient to estimate the standard deviation for future full trials^72^.

Data were analysed in IBM® SPSS® v.29 (*IBM Corp., Armonk, NY, USA, 2022*). Quantitative data were summarised as mean±SD, mean±SE or median(Q1;Q3), based on distribution (Shapiro-Wilk test, histograms, Q-Q plot, skewness/kurtosis); categorical data were presented as absolute/relative frequencies.

Baseline between-group comparisons used unpaired t-tests and Mann-Whitney U tests (quantitative variables) and Fisher’s exact test (categorical variables).

#### Feasibility outcomes

Recruitment, retention and adherence rates were computed with 95% confidence intervals-95%CI. Adverse events were reported as median(Q1;Q3), and absolute/relative frequencies.

#### Preliminary efficacy and effectiveness

Intervention effects were analysed using linear mixed models (restricted maximum likelihood estimation, fixed effects, type III sum of squares) and Friedman tests for continuous data (e.g., min·day^-1^ in light PA, steps·day^-1^, 6MWD, mMRC), and generalised mixed models (restricted maximum likelihood, fixed effects, binomial distribution, logit link, robust standard error), Cochran’s Q and Fisher’s exact tests for categorical data (e.g., participants walking fewer than 5000steps·day^-1^, with impaired 6MWD, or clinically relevant mMRC), with covariance structure (compound symmetry, unstructured, autoregression) selected through likelihood ratio test, Akaike, and Bayesian information criteria. All linear mixed models included a random intercept for participant to account for the repeated-measures structure across time points. For inferential analyses, Bonferroni-adjusted significance thresholds were applied separately for between-group (p<0.017; three comparisons) and within-group analyses (p<0.05; single comparison per group), according to the number of planned comparisons in each analysis and ensuring appropriate control of Type I error.

Efficacy and effectiveness were distinguished in accordance with methodological recommendations^73,74^. Efficacy (per-protocol) analyses included only adherent participants who competed all assessments, i.e., under ideal conditions, and therefore contained no missing/invalid data, whereas effectiveness (intention-to-treat) analyses included all randomised participants, i.e., under usual, real-world conditions, regardless of adherence. Intention-to-treat analyses were conducted using linear mixed models, which accommodate missing data due to attrition under the assumption of data missing at random and therefore did not require imputation^75,76^.

As sensitivity analyses, to account for baseline differences in time from acute COVID-19, lung function and dyspnoea, additional models were run adjusting for time from since acute infection (months), FEV_1_pp, FVCpp and mMRC dyspnoea score as covariates.

## Results

### Participants

**Fig. 3:**
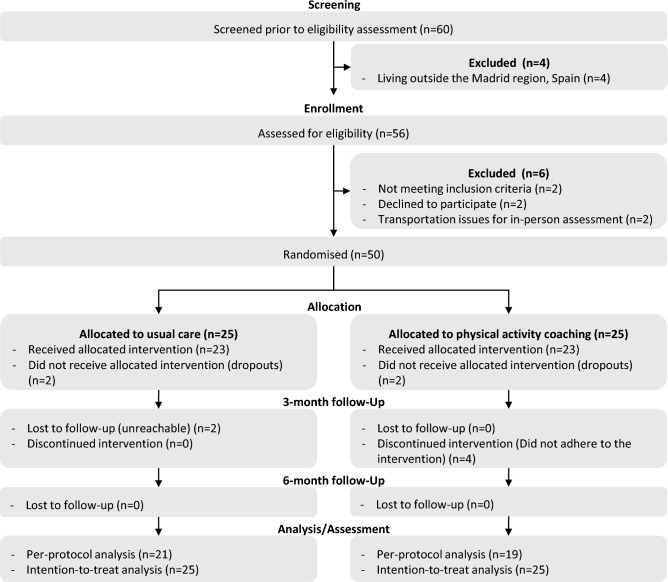
Consolidated Standards of Reporting Trials-CONSORT flow diagram of the physical activity coaching programme for people with Long COVID.

In total, sixty people with Long COVID were screened, fifty-six were eligible and fifty were randomised equally to either the CG or EG (Fig. 3). Forty-six (twenty-three/group) completed the three-month follow-up, while forty (twenty-one CG, nineteen EG) completed the six-month follow-up and adhered to the intervention (Fig. 3).

The intention-to-treat dataset comprised fifty people of 46±11years, 76% female, and 42(26;48)months post-COVID-19 (Table 1). Dyspnoea severity was lower in the EG compared to the CG (mMRC: 1(1;2) vs. 2(2;3); mMRC≥2: 44% vs. 80%) (Table 2). No other significant differences were observed.

**Table 1:** Participants’ baseline characteristics, intention-to-treat dataset, from the physical activity coaching programme for people with Long COVID.

	All	Usual care	PA coaching
**Subjects n**	**50**	**25**	**25**
Age (years) mean±SD	46±11	46±11	47±10
Female/male	38(76)/12(24)	20(80)/5(20)	18(72)/7(28)
BMI (kg·m^-2^) mean±SD	26.6±6.6	28.5±7.6	24.8±4.8
FEV_1_pp mean±SD	99±13	94±11	104±14*
FVCpp mean±SD	95±13	90±10	100±13*
Peak flow pp mean±SD	105±23	103±24	108±23
Time from acute COVID-19 (months) median(Q1;Q3)	42(26;48)	31(24;48)	46(36;48)
Exacerbations median(Q1;Q3)	6(3;12)	7(3;18)	6(4;10)
Exacerbations last 12 months median(Q1;Q3)	2(1;6)	2(1;12)	3(1;4)
Comorbidities^§^ median(Q1;Q3)	1(0;2)	1(0;3)	1(0;2)
Charlson comorbidity index	0(0;1)	0(0;1)	1(0;1)
Charlson comorbidity categories (mild/moderate/severe)	21(42)/3(6)/0(0)	8(32)/3(12)/0(0)	13(52)/0(0)/0(0)
Medications (n)	2(1;5)	2(0;5)	2(1;5)
Medication			
- Antidepressants^§§^	22(44)	11(44)	11(44)
- Analgesics^§§^	14(28)	6(24)	8(32)
- Antihypertensives^§§^	14(28)	6(24)	8(32)
- Short-acting bronchodilators^§§^	11(22)	4(16)	7(28)
- Others^§§^	34(68)	17(68)	17(68)

^§^International Classification of Diseases, 11^th^ Ed.: 22% nervous system diseases, 18% respiratory system and metabolic diseases, 12% circulatory and digestive system diseases, and 10% mental disorders.

^§§^Anatomical Therapeutic Chemical Classification System.

BMI: body mass index; FEV_1_pp: forced expiratory volume in one-second percentage predicted; FVCpp: forced vital capacity percentage predicted; PA: physical activity.

Data are expressed as n(%), unless otherwise reported. *Statistically significant difference between groups at baseline (p-value<0.05).

**Table 2:** Preliminary efficacy and effectiveness of physical activity coaching vs. usual care in people with Long COVID, unadjusted data.

		Per-protocol				Intention-to-treat			
	**Time point**	**Usual care**	**PA coaching**	**MD[95%CI] or RD[95%CI]**	Sig. group*time	**Usual care**	**PA coaching**	**MD[95%CI] or RD[95%CI]**	Sig. group*time
Subjects n Secondary outcomes^#^		**21**	**19**			**25**	**25**		
LPA, min·day^-1^	Baseline	128±14	144±15	-16[-58;26]	p<0.01*	121±13	134±13	-13[-50;24]	p<0.01*
	3 months	105±13†	169±14†	--64[-102;-26]*		100±13	153±12	-53[-89;-18]*	
	6 months	89±14†	174±14†	-85[-125;-45]*		84±13†	158±13	-73[-111;-36]*	
MVPA, min·day^-1^	Baseline	3±1	4±1	-1[-5;2]	p=0.11	3±1	4±1	-1[-4;2]	p=0.11
	3 months	4±2	8±2†	-5[-9;0]		4±2	9±2†	-5[-10;0]	
	6 months	3±2	10±2†	-7[-13;0]		3±2	11±2†	-7[-14;-1]*	
Steps·day^-1^	Baseline	5595±738	6904±776	-1309[-3476;859]	p<0.01*	5369±638	6735±638	-1367[-3182;448]	p<0.01*
	3 months	4772±768	10084±808†	-5312[-7569;-3056]*		4571±711	9203±700†	-4632[-6638;-2626]*	
	6 months	4292±820†	10233±962†	-5941[-8349;-3532]*		4103±770s	9339±753†	-5236[-7402;-3070]*	
Steps·day^-1^≤5000 n(%)	Baseline	10(48)	6(32)	16[-15;47]	p<0.01*	13(52)	9(36)	16[-12;44]	p=0.10
	3 months	12(57)	2(11)	51[25;77]*		12(48)	4(16)	41[15;68]*	
	6 months	13(62)	3(16)	51[23;78]*		13(52)	4(16)	44[17;71]*	
Sedentary time, min·day^-1^	Baseline	678±24	668±25	10[-61;81]	p<0.01*	678±22	661±22	17[-47;80]	p<0.01*
	3 months	688±26	612±27†	76[-1;153]		688±25	616±24†	71[2;141]*	
	6 months	697±25	594±26†	104[30;177]*		697±24	599±23†	98[31;165]*	
6MWD, m	Baseline	445±25	487±27	-42[-116;32]	p<0.01*	450±23	495±23	-45[-111;22]	p<0.01*
	3 months	423±26	542±27†	-119[-194;-44]*		427±24	546±24†	-119[-187;-51]*	
	6 months	422±25	558±26†	-136[-210;-62]*		426±24	562±24†	-136[-203;-69]*	
6MWD≤70pp n(%)	Baseline	9(43)	10(53)	-10[-42;22]	p<0.01*	10(40)	11(44)	-4[-32;24]	p=0.02*
	3 months	13(62)†	3(16)†	46[19;73]*		13(52)†	5(20)†	40[14;67]*	
	6 months	13(62)†	2(11)†	51[26;77]*		13(52)†	4(16)†	45[19;70]*	
1minSTS, reps	Baseline	20±3	28±3	-7[-15;0]	p=0.29	21±2	26±2	-6[-12;1]	p=0.20
	3 months	22±3	31±3†	-9[-17;-2]*		22±2	30±2†	-7[-14;-1]	
	6 months	21±3	32±3†	-11[-18;-3]*		22±2	31±2†	-9[-15;-2]*	
1minSTS≤70pp n(%)	Baseline	16(76)	9(47)	29[-1;59]	p=0.26	18(72)	14(56)	16[-11;43]	p=0.25
	3 months	13(62)	8(42)	20[-12;51]		13(52)	11(44)	7[-22;36]	
	6 months	14(67)	6(32)	35[5;65]*		14(56)	9(36)	22[-7;51]	
QMVC, kgf	Baseline	20±2	21±2	-1[-7;5]	p=0.02	21±2	21±2	0[-5;6]	p=0.02
	3 months	19±2	24±2†	-5[-11;0]		20±2	23±2	-3[-8;2]	
	6 months	18±2	23±2†	-5[-11;0]		19±2	23±2	-3[-8;2]	
QMVC≤70pp n(%)	Baseline	4(19)	2(11)	3[-21;27]	p=0.32	4(16)	3(12)	4[-16;24]	p=0.38
	3 months	4(19)	3(16)	9[-14;31]		4(16)	3(12)	5[-17;26]	
	6 months	5(24)	2(11)	19[-3;40]		5(20)	2(8)	14[-7;35]	
Handgrip, kgf	Baseline	24±2	23±3	2[-6;9]	p=0.13	25±2	23±2	2[-4;8]	p=0.10
	3 months	24±2	25±2	-1[-8;5]		25±2	26±2†	-1[-7;5]	
	6 months	24±2	26±2†	-2[-9;5]		25±2	26±2†	-2[-8;4]	
Handgrip ≤70pp n(%)	Baseline	10(48)	11(58)	-10[-42;21]	p=0.06	11(44)	13(52)	-	p=0.03
	3 months	12(57)	7(37)	20[-11;52]		12(48)	7(28)†	-	
	6 months	13(62)	6(32)†	30[0;61]*		13(52)	6(24)†	-	
PImax, cmH2O	Baseline	65±5	69±5	-4[-19;11]	p=0.04	68±5	66±5	2[-11;15]	p=0.01*
	3 months	60±5	72±5	-12[-26;3]		62±5	69±5	-6[-19;6]	
	6 months	57±4	74±5	-17[-30;-4]*		59±4†	71±4	-13[-24;-1]*	
PImax≤70pp n(%)	Baseline	15(71)	12(63)	8[-22;38]	p=0.15	18(72)	17(68)	4[-22;30]	p=0.21
	3 months	17(81)	10(53)	28[-1;57]		17(68)	14(56)	19[-8;45]	
	6 months	19(91)	10(53)	38[11;64]*		19(76)	14(56)	29[5;52]*	
PEmax, cmH2O	Baseline	73±7	85±8	-12[-34;10]	p=0.04	79±7	82±7	-3[-22;16]	p=0.01*
	3 months	79±6	103±6†	-25[-41;-8]*		83±5	99±5†	-17[-32;-1]*	
	6 months	78±6	109±6†	-31[-48;-14]*		82±6	105±5†	-23[-39;-8]*	
PEmax≤70pp n(%)	Baseline	19(91)	17(90)	1[-18;20]	p<0.01*	22(88)	23(92)	-4[-21;13]	p<0.01*
	3 months	21(100)	13(68)	32[8^55^*		21(84)	17(68)	26[6;45]*	
	6 months	21(100)	11(58)†	42[17;68]*		21(84)	15(60)†	34[13;56]*	
Dyspnoea mMRC	Baseline	2(2;3)	1(1;2)	1[0;1]	p<0.01*	2(2;3)	1(1;2)	1[0;1]*	p=0.01*
	3 months	2(1;3)	1(0;1)†	1[1;2]*		2(1;3)	1(0;1)†	1[1;2]*	
	6 months	2(1;3)	1(0;1)†	1[1;2]*		2(1;3)	1(1;1)†	1[1;2]*	
mMRC≥2 n(%)	Baseline	17(81)	9(47)	34[5;63]*	p<0.01*	20(80)	11(44)	36[10;62]*	p<0.01*
	3 months	15(71)	1(5)†	66[43;90]*		15(60)	1(4)†	66[45;88]*	
	6 months	15(71)	0(0)†	71[50;93]*		15(60)	0(0)†	71[50;92]*	
Fatigue FACIT-FS	Baseline	17(12;25)	19(10;26)	1[-6;8]	p<0.01*	17(12;25)	17(9;23)	2[-5;8]	p<0.01*
	3 months	15(12;26)	27(24;37)†	-12[-18;-6]*		15(12;26)	27(22;35)†	-11[-17;-6]*	
	6 months	16(12;27)	37(32;44)†	-19[-24;-14]*		16(12;27)	37(32;44)†	-19[-24;-13]*	
FACIT-FS≤43 n(%)	Baseline	21(100)	19(100)	-	p<0.01*	24(96)	25(100)	-4[-12;5]	p<0.01*
	3 months	21(100)	18(95)†	-		21(84)	22(88)	4[-5;14]	
	6 months	21(100)	13(68)†	-		21(84)	16(64)†	30[10;51]*	
PEM DSQ n(%)	Baseline	19(91)	18(95)	-4[-21;12]	p=0.16	23(92)	24(96)	-4[-17;9]	p=0.15
	3 months	18(86)	13(68)†	17[-9;44]		18(72)	17(68)	12[-12;36]	
	6 months	20(96)	12(63)†	32[8;56]*		20(80)	15(60)†	30[8;52]*	
ME/CFS DSQ n(%)	Baseline	11(52)	14(74)	-21[-51;9]	p<0.01*	13(52)	19(76)	-24[-50;2]	p<0.01*
	3 months	15(71)	4(21)†	50[23;78]*		15(60)	5(20)†	50[24;76]*	
	6 months	15(71)	0(0)†	71[50;93}*		15(60)	1(4)†	67[46;89]*	
Anxiety HADS-A	Baseline	7(4;11)	10(7;14)	-2[-5;1]	p=0.38	7(4;11)	10(7;14)	-2[-4;1]	p=0.41
	3 months	6(4;12)	10(4;12)	-1[-4;3]		6(4;12)	10(5;14)	-1[-4;2]	
	6 months	7(3;12)	11(5;14)	-2[-5;1]		7(3;12)	11(5;14)	-2[-5;1]	
HADS-A≥8 n(%)	Baseline	9(43)	14(74)	-31[-61;-1]*	p=0.34	11(44)	18(72)	-28[-55;-1]*	p=0.26
	3 months	9(43)	12(63)	-20[-52;11]		9(36)	14(56)	-16[-45;13]	
	6 months	10(48)	12(63)	-16[-47;16]		10(40)	14(56)	-11[-40;18]	
Depression HADS-D	Baseline	10(6;12)	11(4;12) 9(4;12)	1[-3;4]	p=0.44	10(6;12)	11(5;15)	0[-2;3]	p=0.56
	3 months	8(6;12)	9(6;13)	0[-3;3]		8(6;12)	10(6;13)	0[-3;2]	
	6 months	8(4;11)†		-1[-4;2]		8(4;11)†	9(6;12)	-1[-3;2]	
HADS-D≥8 n(%)	Baseline	14(67)	11(58)	-9[-22;40]	p=0.58	17(68)	16(64)	4[-23;31]	p=0.55
	3 months	12(57)	12(63)	-6[-37;25]		12(48)	16(64)	-11[-40;18]	
	6 months	11(52)	11(58)	-6[-37;26]		11(44)	14(56)	-7[-37;23]	
EQ-5D-5L	Baseline	40(30;63)	40(35;55)	4[-6;14]	p<0.01*	40(30;63)	40(30;55)	3[-7;14]	p<0.01*
	3 months	40(33;45)	60(50;65)†	-20[-28;-12]*		40(33;45)	60(50;65)†	-21[-29;-13]*	
	6 months	40(30;50)	60(60;70)†	-24[-32;-16]*		40(30;70)	65(60;70)†	-25[-33;-18]*	
EQ-5D-5L≤threshold n(%)	Baseline	19(91)	19(100)	-10[-24;5]	p<0.01*	22(88)	25(100)	-12[-26;2]	p<0.01*
	3 months	20(95)	17(90)	6[-11;23]		20(80)	20(80)	7[-10;24]	
	6 months	21(100)	15(79)	21[0;42]*		21(84)	18(72)†	22[3;40]*	

Data are reported as mean±SE or median(Q1;Q3), unless otherwise stated.

Risk difference represents the absolute difference in proportions between groups.

1minSTS: 1-min sit-to-stand test; 6MWD: 6-min walking distance; DSQ: DePaul symptom questionnaire; EQ-5D-5L: European quality of life - 5 dimensions - 5 levels; FACIT-FS: functional assessment of chronic illness therapy - fatigue; HADS: hospital anxiety and depression scale; LPA: time spent in light physical activity; MD: mean difference; ME/CFS: myalgic encephalomyelitis/chronic fatigue syndrome; mMRC: modified Medical Research Council; MVPA: time spent in moderate-to-vigorous physical activity; PImax: maximal inspiratory pressure; PEM: post-exertional malaise; PEmax: maximal expiratory pressure; QMVC: quadriceps muscle voluntary contraction; RD: risk difference.

*Statistically significant p-value <0.017 between groups, †Statistically significant p-value <0.05 within each group.

### Feasibility: Recruitment, retention, adherence and adverse events

All feasibility criteria were met. The recruitment rate was 89% (95%CI 79-95%) and retention was 92% (95%CI 81-97%). In the EG, 100% (95%CI 86-100%) attended ≥80% of planned sessions, while 83% (95%CI 63-93%) met their weekly activity targets ≥80% of the time. No adverse events related to the intervention were reported, and there were no significant between-group differences (e-Table 2).

### Preliminary effectiveness of physical activity coaching programme vs. usual care

#### Physical activity and sedentary behaviour

**Fig. 4:**
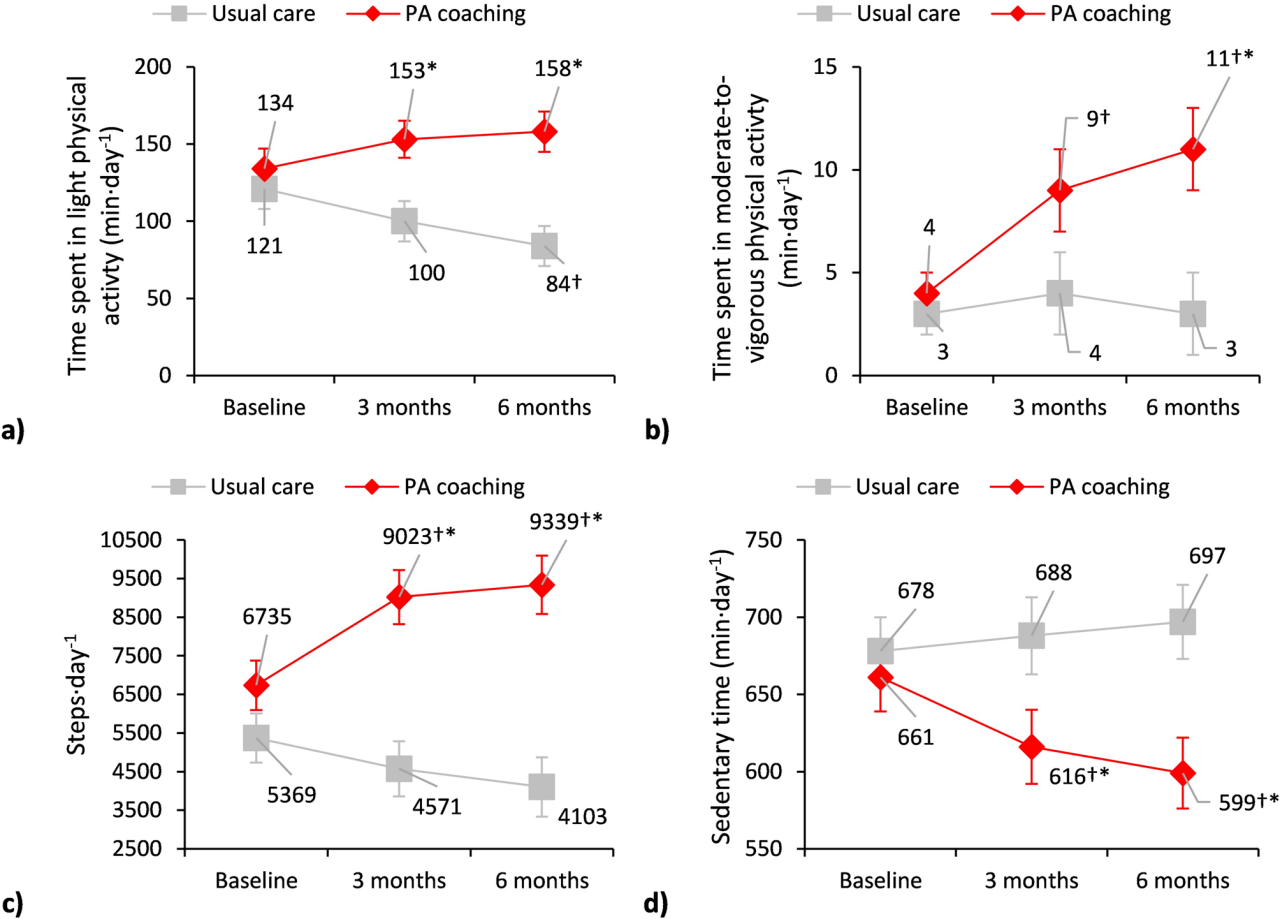
Preliminary effectiveness of physical activity coaching vs. usual care in people with Long COVID on physical activity and sedentary behaviour measures. **a**) Time spent in light physical activity: between-group differences at 3 months, +53[18;89] min·day^-1^, and 6 months: +73[36;111] min·day^-1^. **b**) Time spent on moderate-to-vigorous physical activity: between group differences at 6 months, +7[1;14] min·day^-1^. **c**) Steps·day^-1^: between group difference at 3 months, +4632[2626;6638], and 6 months, +5236[3070;7402]. **d**) Sedentary time: between-group difference at 3 months, -71[-141;-2] min·day^-1^, and 6 months, -98[-165;-31] min·day^-1^. Data are presented as mean±SE. †Statistically significant p-value <0.05 within each group; *Statistically significant p-value <0.017 between groups

The EG demonstrated significant improvements at three-months follow-up, increasing time spent in moderate-to-vigorous PA (+5[1;9]min·day^-1^) and daily steps (+2468[1426;3509]), while decreasing sedentary time (-45[-86;-4]min·day^-1^). These improvements were maintained at six-month follow-up (moderate-to-vigorous PA: +7[2;12]min·day^-1^; step·day^-1^: +2604[1254;3953]; sedentary time: -62[-106;-18]min·day^-1^). In contrast, the CG reduced time spent in light PA at six-month follow-up (-36[-64;-8]min·day^-1^), reflecting a deterioration over time (Table 2, Fig. 4). Moreover, the EG and CG differed significantly in time spent in light PA, daily steps and sedentary behaviour both at three- (light PA: +53[18;89]min·day^-1^; steps·day^-1^: +4632[2626;6638]; sedentary time: -71[-141;-2]min·day^-1^) and six-month follow-up (light PA: +73[36;111]min·day^-1^; steps·day^-1^: +5236[3070;7402]; sedentary time: -98[-165;-31]min·day^-1^), as well as moderate-to-vigorous PA at six-month follow-up (+7[1;14]min·day^-1^) (Table 2, Fig. 4). Additionally, the proportion of participants walking fewer than 5000steps·day^-1^ decreased in the EG, while remaining unchanged in the CG (from baseline to six-month follow-up: 36% to 16% vs. 52% to 52%) (Table 2).

#### Functional capacity, peripheral muscle strength, symptoms and health-related quality of life

The EG improved functional capacity (6MWD: +51[24;78]m; 1minSTS: +4[1;6]reps), dyspnoea (mMRC: -1[-1;0]), fatigue (FACIT-FS: +12[8;15]) and HRQoL (EQ-5D-5L: +20[11;29]) at three-month follow-up. These improvements were sustained at six-month follow-up. By the end of follow-up, the proportion of people affected by impaired functional capacity (6MWD: 44% to 16%), dyspnoea (44% to 0%), fatigue (100% to 64%), post-exertional malaise (96% to 60%), myalgic encephalomyelitis/chronic fatigue syndrome (76% to 4%) and impaired HRQoL (100% to 72%) had decreased (Table 2). Conversely, the CG was more functionally impaired (6MWD: 40% to 52%) and presented persistent symptoms (dyspnoea: 60%; fatigue: 84%; post-exertional malaise: 80%; myalgic encephalomyelitis/chronic fatigue syndrome: 60%) and impaired HRQoL (84%) by the end of follow-up (Table 2). Significant differences between the EG and CG were found in functional capacity, dyspnoea, fatigue ad HRQoL at three- (6MWD: +119[51;187]m; mMRC: -1[-2;-1]; FACIT-FS: +11[6;17]; EQ-5D-5L: +21[13;29]) and six-month follow-up (6MWD: +136[69;203]m; 1minSTS: +9[2;15]reps; mMRC: -1[-2;-1]; FACIT-FS: +19[13;24]; EQ-5D-5L: +25[18;33]) (Table 2).

**Fig. 5:**
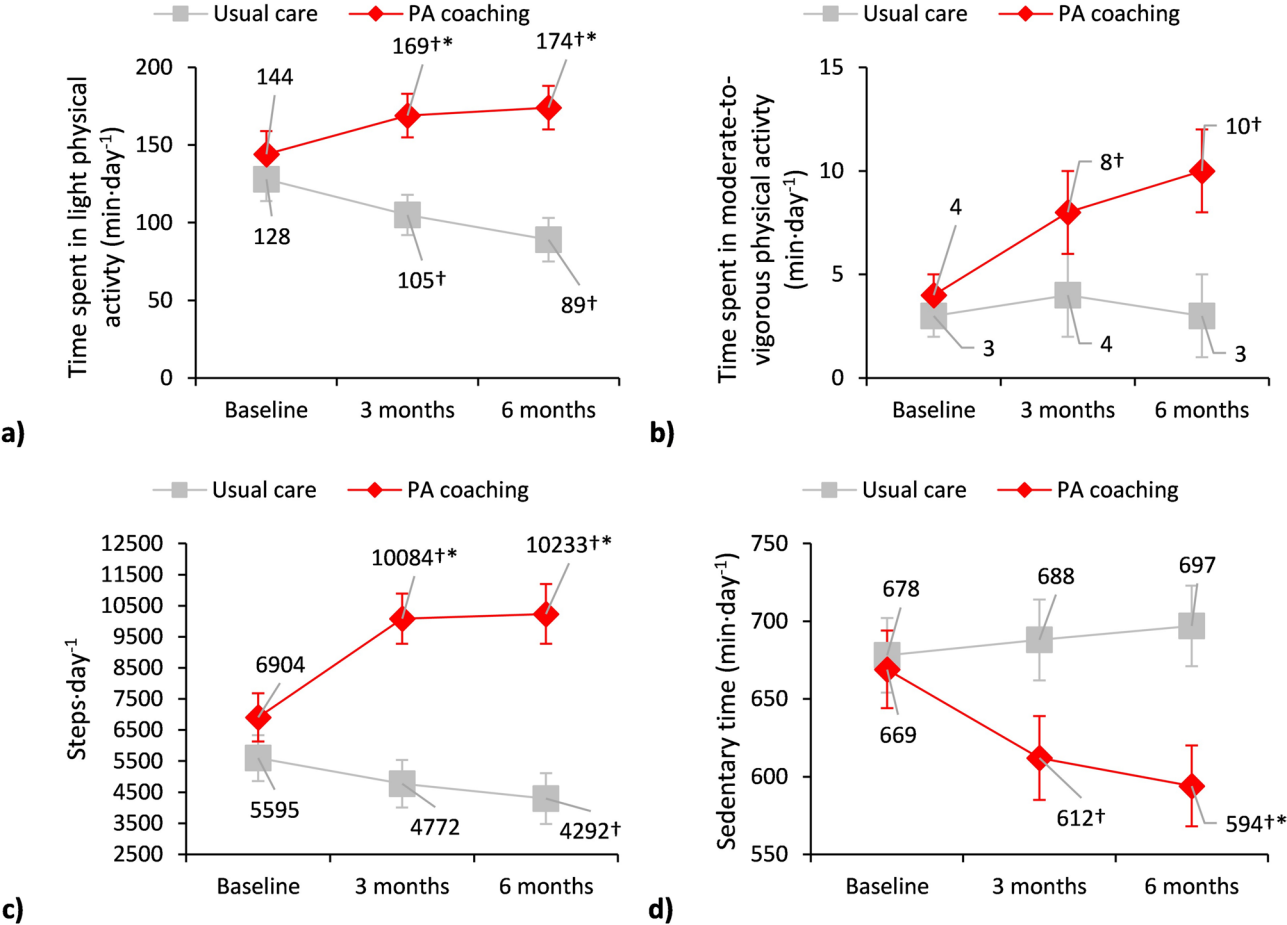
Preliminary efficacy of physical activity coaching vs. usual care in people with Long COVID on physical activity and sedentary behaviour measures. **a**) Time spent in light physical activity: between-group differences at 3 months, +64[26;102] min·day^-1^, and 6 months: +85[45;125] min·day^-1^. **b**) Time spent on moderate-to-vigorous physical activity. **c**) Steps·day^-1^: between group difference at 3 months, +5312[3056;7569], and 6 months, +5941[3532;8349]. d) Sedentary time: between-group difference at 6 months, -104[-177;-30] min·day^-1^. Data are presented as mean±SE. †Statistically significant p-value <0.05 within each group; *Statistically significant p-value <0.017 between groups.

Further details on the results for the per-protocol dataset are reported in Table 2, Fig. 5 and Additional files 1-3.

Sensitivity analyses adjusting for time from acute COVID-19, lung function and dyspnoea yielded results which were consistent in direction, although of slightly lower magnitude but still clinically relevant, compared with the unadjusted models (Additional file 4).

## Discussion

This randomised pilot trial assessed the feasibility and preliminary efficacy and effectiveness of a remotely delivered, one-to-tone, PA coaching programme vs. usual care in people with Long COVID. All feasibility criteria were met, with high recruitment, retention and adherence, and no serious adverse events were reported. This intervention effectively increased PA and reduced sedentary behaviour, and these changes were maintained or further improved up to three-months after intervention. Functional capacity, dyspnoea, fatigue, post-exertional malaise, and HRQoL also improved, although psychological outcomes did not.

The PA coaching programme integrated behavioural contents, i.e., self-monitoring, tailored feedback, and goal setting/review, responsive to the individual needs, and results support its feasibility, acceptability and safety in people with Long COVID. This intervention was person-centred and symptom-responsive, prompting PA behaviour adaptation and sustained engagement, in line with current disease management recommendations, which prioritise pacing and symptom-responsive strategies^24^.

Findings are consistent with previous studies showing feasibility of PA interventions in this population when remotely delivered. Indeed, remote programmes reported higher recruitment (86%, 95%CI 82-90%), retention (94%, 95%CI 91-98%) and adherence (85%, 95%CI 79-92%)^77–79^ than outpatient interventions (recruitment 68%, 95%CI 63-72%; retention 81%, 95%CI 76-85%; adherence 72%, 95%CI 67-77%)^80^, However, previous trials often show limited or inconsistent efficacy, possibly because of intervention contents (exercise vs. PA-behaviour driven), intervention delivery (outpatient vs. remote), intervention length (short vs. long), or lack of consideration in baseline between-group differences.

Previous trials assessing structured exercise (to achieve 150min·week^-1^ of moderate PA) or tele-counselling yielded mixed results, often lacking between-group differences (experimental vs. control) although with significant PA changes over time^77,78^. In contrast, this behaviour-centred PA intervention vs. usual care produced statistically and clinically relevant PA and sedentary behaviour changes, suggesting its adaptability to and tolerability by people with Long COVID, with fluctuating symptoms, and potentially promoting sustained PA engagement^81,82^. Indeed, included behaviour-change contents, e.g., self-monitoring and goal setting/review, known as moderators of PA coaching intervention effectiveness in people with chronic respiratory diseases^28^, may have supported self-regulation and day-to-day adjustment of activity^83^. PA could be adjusted to daily symptom burden, and may have reduced post-exertional exacerbation risk^24^, therefore prompting safer PA engagement (something not always possible in exercise-based interventions). Graded goal setting and progressive PA targets remind activity pacing strategies, which have shown benefits in people with myalgic encephalomyelitis/chronic fatigue syndrome, a condition prevalent in Long COVID (64% of participants). In this population, graded activity approaches, with progressive activity increase while avoiding overexertion, rather than a pure pacing approaches, with maintained activity within a intensity range, can improve function without symptom exacerbations^84^. Adjusting goals to symptom burden while increasing activity quantity and intensity, our intervention may have reproduced this approach, likely reduced post-exertional symptom exacerbations, and prompted safe PA engagement.

Furthermore, remote delivery may have reduced PA barriers, e.g., travel fatigue, time and financial constraints, limited access to rehabilitation services^5,85^, further supporting physically active behaviour; and a longer intervention (i.e., twelve-weeks vs. six-weeks^77^) may have provided sufficient time for participants to understand behavioural contents (intervention vs. participant action-response), perform, or include behaviour-change in their daily routine^86–88^.

This PA behavioural intervention also produced significant changes in functional capacity beyond established minimal clinically important thresholds^42,89,90^. PA interventions yield better outcomes when activity is supported through real-world, community-integrated behavioural approaches (e.g., urban training), rather than exclusively outpatient centre-based exercise programmes^91^. On one side, improvements in symptoms of dyspnoea, fatigue and post-exertional malaise may have reduced task limitation and enhanced performance^42,92^. At the same time, improvements in peripheral muscle strength (e.g., QMVC, handgrip, PEmax) may have also supported physiological adaptations. On the other side, increased self-efficacy and self-confidence, reduced fear-avoidance, improved pacing and graded activity implementation may have supported willingness to exert and physical capacity^93^. However, as this was a PA behavioural intervention, and pilot nature of study design, these findings should be considered preliminary and exploratory. Future fully powered trials should assess behavioural vs. physiological mechanisms more accurately.

Cognitive behavioural therapy-based interventions have shown improvements in physical function, fatigue, dyspnoea and psychological distress in Long COVID^79,80^, although none have specifically addressed PA or sedentary behaviour outcomes. This PA coaching intervention improved physical- and symptom-based outcomes, as well as HRQoL, but did not change symptoms of anxiety or depression. In Long COVID, psychological distress may be driven not only by physical limitations but also by impaired self-esteem and identity loss, frustration, social isolation, feelings of being misunderstood or burdensome^5,85,94,95^. Such psychosocial factors are unlikely to be addressed exclusively through PA-focused interventions. Indeed, specialised psychological support, particularly when appropriately delivered (e.g., one-to-one vs. group, face-to-face vs. remote), can improve mental health^96^. Furthermore, psychological therapy within individualised PA interventions has shown benefits for both PA and psychological distress in people with chronic respiratory diseases, and comorbid symptoms of anxiety and depression^97^. Future trials should therefore consider integrating PA behavioural interventions with specialised psychological support in order to address patient mental health needs.

This study is among the first to assess a remote one-to-one PA coaching programme vs. usual care in people with Lond COVID. The intervention was informed by a systematic review with meta-analysis^28^ and co-designed with a patient advocacy group. Patient input shaped recruitment strategy, implemented materials, and intervention format, following current recommendations for best practice in feasibility and pilot trials^33^. Objective, continuous PA and sedentary behaviour monitoring supported safety and informed decision-making on PA volume and intensity, while remote delivery improved accessibility, addressing known PA barriers in people with Long COVID^22,24^.

However, several limitations should be acknowledged. Baseline between-group differences were observed in time since acute infection, lung function and dyspnoea. Although lung function (e.g., FEV_1_) shows a weak-to-moderate positive association with PA in people with chronic respiratory diseases (e.g., COPD), it explains only a modest proportion of variability in PA behaviour^11^. Furthermore, lung function was well preserved in both groups (i.e., FEV_1_pp: CG: 94±11, vs. EG: 104±14), suggesting a limited influence on PA measures. In contrast, participants in the EG had a longer time since acute COVID-19, and may therefore have reached a more stable phase of the disease. Natural history of the disease could have partially contributed to PA engagement, regardless of the intervention. However, recovery trajectories are heterogenous and often non-linear in Long COVID^98,99^. Indeed, despite differences in time since acute infection, baseline physical function (functional capacity, peripheral muscle strength) and symptom burden (fatigue, post-exertional malaise, anxiety and depression) did not differ between groups. Lower dyspnoea severity in the EG may have also favoured a more physically active behaviour^7,100^. Sensitivity analyses adjusting for time since acute infection, lung function and dyspnoea yielded results consistent in direction with the unadjusted analyses, suggesting that intervention effects were not solely attributable to baseline differences. Nevertheless, future trials should employ stratified randomisation to minimise differences in potential prognostic factors, including time since infection and symptom severity. Furthermore, participants were mostly female and middle-aged (76% female; 46±11 years). While this distribution reflects epidemiology of Long COVID, which is more frequently reported in females and adults aged 40-54 years or ≥65 years^101–103^, it may limit generalisability of findings to younger and older, or male populations. Participants potentially presented certain digital literacy, which may have supported intervention acceptability. Future studies should integrate support strategies for older population or populations with limited digital access.

Usual care might have been contaminated, as all participants received an activity tracker on the first visit before randomisation, and then were indicated to submit weekly PA reports after randomisation (i.e., randomisation process was conducted one-week after the first visit for PA assessment). This ensured equivalent measurement conditions between groups, although it may have also introduced an element of self-monitoring. Self-monitoring is indeed a key behaviour change technique within PA interventions^28^, although it is unlikely to produce sustained behaviour change unless integrated within comprehensive behavioural interventions addressing all determinants of behaviour, namely capability, opportunity and motivation^29^. Although previous studies support that activity trackers may encourage more physically active behaviour^104–106^, their effect is maximised when combined with additional coaching strategies (e.g., personalised feedback, goal setting and review, and motivation interviewing through a collaborative, person-centred approach)^105^. This minimal intervention may therefore limit the interpretation of findings. However, any behaviour change resulting from self-monitoring in the CG could have attenuated between-group post-intervention differences, and underestimated true effect size of PA coaching programme. Furthermore, the use of usual care rather than an attention-matched control intervention may have introduced attention or expectation bias, which could have influenced patient-reported outcomes. However, this intervention represents current standard care for chronic conditions and Long COVID management^37,38^, and was reviewed in collaboration with a patient advocacy group, which considered it acceptable and representative of real-world care.

The custom-designed inertial measurement unit algorithms were validated in healthy adults under controlled laboratory and overground conditions rather than in people with Long COVID. Although inertial systems approaches have demonstrated excellent accuracy for activity classification (i.e., 97%) and step detection (i.e., 3.5% mean absolute error), and have shown robust validity and reliability in other clinical populations (e.g., chronic respiratory diseases)^39,40^, their performance in people with Long COVID has not yet been formally established. In this population, gait characteristics, e.g., reduced cadence, shuffling, increased step-to-step variability may affect step detection accuracy when algorithms have been trained on able-bodied data^107^. Nevertheless, inertial measurement unit-based data processing implemented using MATLAB represents a well-established, transparent and reproducible approach for objective PA assessment, previously validated against criterion measures (e.g., video-based systems) and medical devices (e.g., triaxial accelerometers, Actigraph®), and demonstrated sensitivity at low walking speeds^108^. Furthermore, as the same device, wear location and data processing were consistent across both groups, any systematic measurement error would be expected to be non-differential and therefore bias effect estimates minimal or null. Future definitive trials should include population-specific validation and test-retest reliability assessments to confirm accuracy and longitudinal stability in people with Long COVID.

Performance bias could not be fully prevented, as blinding of participants and physiotherapists was not feasible due to the behavioural nature of the intervention; however, outcome assessors conducting clinical and functional assessments were blinded to group allocation. This may have influenced interaction dynamics and, potentially, patient-reported outcomes.

While most participants reported post-exertional malaise, the sample did not exclusively consist of physically inactive individuals. Future research should refine inclusion criteria to target those at risk of PA decline. Recruitment from a single patient advocacy group may have introduced selection bias, potentially favouring participants who were more motivated, health-literate or engaged with self-management, thus limiting generalisability.

Finally, although physical and symptom-based outcomes improved, no significant changes were observed in symptoms of anxiety and depression. As discussed above, this likely reflects the lack of dedicates psychological support within the intervention. Future iterations should integrate such support to address mental health needs.

Therefore, future full-scale trials should include stratified participant recruitment and randomisation based on potential confounders (e.g., age, sex) and prognostic factors (e.g., time since infection and symptom severity); strategies to minimise usual care contamination; population-specific validation of objective outcome measurement devices; and intervention adaptations (including specialised psychological support, where needed) to accommodate individual symptoms. Furthermore, scalability may be enhanced by optimising remote delivery, tailoring support to different levels of health and digital literacy, and education, and integrating the intervention within existing care pathways (e.g., primary care screening to identify target population, with referral to secondary or tertiary care or community services, where professionals trained in PA behavioural interventions are available). Addressing these factors will be essential to support wider implementation and generalisability.

## Conclusions

This pilot trial showed that a remotely delivered, one-to-one PA coaching programme is feasible, safe, and may improve PA, sedentary behaviour, functional capacity, symptoms and HRQoL in people with Long COVID. These findings support progression to a fully powered trial, with adjustments to participant selection, randomisation methods (e.g., stratified recruitment and randomisation), and usual care without contamination (e.g., PA tracker provision, PA reports). Furthermore, future studies should also explore integrating psychological support and leveraging digital technologies (e.g., objectively measured PA and sedentary behaviour monitoring, remote delivery) to develop personalised, scalable and engaging interventions.

## Supplementary Information

Supplementary Information 1.

Supplementary Information 2.

Supplementary Information 3.

Supplementary Information 4.

Supplementary Information 5.

Supplementary Information 6.

Supplementary Information 7.

## Data Availability

The data supporting this study are available from the corresponding author upon reasonable request.

## Abbreviations

1minSTS: One-minute sit-to-stand
6MWT/6MWD: Six-minute walking test/distance
AMACOP: Asociación Madrileña de COVID Persistente
BCT: Behaviour-change technique
CG: Control group
CONSORT: Consolidated Standards of Reporting Trials
DSQ: DePaul Symptom Questionnaire
EG: Experimental group
EQ-5D-5L: European Quality of Life-Five Dimensions-Five Levels
FACIT-FS: Functional Assessment of Chronic Illness Therapy-Fatigue
FEV_1_: Forced expiratory volume in one second
FEV_1_pp: Forced expiratory volume in one second percentage predicted
FVC: Force vital capacity
FVCpp: Force vital capacity percentage predicted
HADS: Hospital Anxiety and Depression Scale
HRQoL: Health-related quality of life
mMRC: Modified Medical Research Council
PA: Physical activity
QMVC: Quadriceps maximal voluntary contraction
PEmax: Maximal expiratory pressure
PImax: Maximal inspiratory pressure
TIDieR: Template for intervention description and replication
