# Supplementary material for: Physical activity coaching programme for people with Long COVID: a pilot randomised clinical trial
Source: Sci Rep. 2026 Mar 24;16:14820. doi: 10.1038/s41598-026-44806-9 (PMC13168267; doi:10.1038/s41598-026-44806-9)
Supplement: Supplementary file 1 — Supplementary Information 1. [file 41598_2026_44806_MOESM1_ESM.docx]

### **Preliminary efficacy of physical activity coaching programme vs. usual care on physical activity, sedentary behaviour and other outcomes**

The EG showed significantly increased time spent in light PA (+25[2;49]min·day^-1^), moderate-to-vigorous PA (+4[1;7]min·day^-1^) and daily steps (+3180[2233;4128]), and reduced sedentary time (-56[-102;-11]min·day^-1^) at three-month follow-up. These changes were maintained or further improved at six-month follow-up (light PA: +30[1;61]min·day^-1^; moderate-to-vigorous PA: +6[1;11]min·day^-1^; step·day^-1^: +3329[1967;4691]; sedentary time: -75[-124;-26]min·day^-1^). Conversely, the CG decreased PA, reducing time spent in light PA (-23[-45;-1]min·day^-1^ at three-month follow-up; -39[-67;-10]min·day^-1^ at six-month follow-up) and daily steps (-1303[-2599;-7] at six-month follow-up). Furthermore, between-group differences were observed at three- (light PA: +64[102;26]min·day^-1^; steps·day^-1^: +5312[7569;3056]) and six-month follow-up (light PA: +85[125;45]min·day^-1^; steps·day^-1^: +5941[8349;3532]; sedentary time: -104[-30;-177]min·day^-1^) (TABLE 2, FIGURE 4). The proportion of participants walking fewer than 5000steps·day^-1^ decreased in the EG (32% to 16%), while increased in the CG (48% to 62%) from baseline to six-month follow-up (TABLE 2).

The EG significantly improved functional capacity (6MWD: +54[23;85]m; 1minSTS: +4[1;6]reps), dyspnoea (mMRC:-1[-1;0]), fatigue (FACIT-FS: +11[8;15]) and HRQoL (EQ-5D-5L: +20[15;25]) at three-month follow-up, with these changes maintained or further improved at six-months follow-up. By the end of follow-up, the EG reduced the proportion of participants with impaired functional capacity (6MWD: 53% to 11%), dyspnoea (47% to 0%), fatigue (100% to 68%), post-exertional malaise (95% to 63%) and myalgic encephalomyelitis/chronic fatigue syndrome (74% to 0%) (TABLE 2). In contrast, the CG increased the proportion of people with impaired functional capacity (6MWD: 43% to 62%), and reporting persistent symptoms (dyspnoea: 71%; fatigue: 100%; post-exertional malaise: 96%; myalgic encephalomyelitis/chronic fatigue syndrome: 91%) and impaired HRQoL (100%) at six-month follow-up. Significant between-group differences were found at three- (6MWD: +119[44;194]m: mMRC: -1[-2;-1]; FACIT-FS: +12[6;18]; EQ-5D-5L: +20[12;28]) and six-month follow-up (6MWD: +136[62;210]m: mMRC: -1[-2;-1]; FACIT-FS: +19[14;24]; EQ-5D-5L: +24[16;32]) (TABLE 2).
