# Supplementary material for: Physical activity coaching programme for people with Long COVID: a pilot randomised clinical trial
Source: Sci Rep. 2026 Mar 24;16:14820. doi: 10.1038/s41598-026-44806-9 (PMC13168267; doi:10.1038/s41598-026-44806-9)
Supplement: Supplementary file 2 — Supplementary Information 2. [file 41598_2026_44806_MOESM2_ESM.docx]

### **e-TABLE 1. Participants’ baseline characteristics, per-protocol dataset, from the physical activity coaching programme for people with Long COVID**

|  | All | Usual care | PA coaching |
| --- | --- | --- | --- |
| **Subjects n** | **40** | **21** | **19** |
| Age (years) mean±SD | 47±11 | 46±11 | 47±10 |
| Female/male | 31(77)/9(23) | 17(81)/4(19) | 14(74)/5(26) |
| BMI (kg·m^-2^) mean±SD | 26.4±5.7 | 27.4±6.2 | 25.4±5.1 |
| FEV_1_pp mean±SD | 98±14 | 93±11 | 102±15* |
| FVCpp mean±SD | 93±13 | 88±10 | 99±14* |
| Time from acute COVID-19 (months) median(Q1;Q3) | 42(27;48) | 30(24;48) | 47(41;48)* |
| Exacerbations median(Q1;Q3) | 6(3;12) | 6(3;21) | 6(4;10) |
| Exacerbations last 12 months median(Q1;Q3) | 2(1;5) | 2(2;9) | 2(1;3) |
| Comorbidities^§^ median(Q1;Q3) | 2(1;3) | 2(1;3) | 2(1;2) |
| Charlson comorbidity index | 0(0;1) | 0(0;1) | 0(0;1) |
| Charlson comorbidity categories (mild/moderate) | 16(40)/3(8) | 7(33)/3(14) | 9(47)/0(0) |
| Medications median(Q1;Q3) | 2(0;5) | 2(0;6) | 3(1;5) |
| Medication |  |  |  |
| - Antidepressants^§§^ | 19(48) | 10(48) | 9(47) |
| - Analgesics^§§^ | 13(33) | 6(29) | 7(37) |
| - Antihypertensives^§§^ | 12(30) | 4(19) | 8(42) |
| - Short-acting bronchodilators^§§^ | 10(25) | 3(14) | 7(37) |
| - Others^§§^ | 28(70) | 4(67) | 14(74) |
| Data are expressed as n(%), unless otherwise reported. *Statistically significant p-value<0.05.  ^§^International Classification of Diseases, 11^th^ Ed.: 28% nervous system diseases, 23% metabolic diseases, 20% respiratory system diseases, 13% circulatory and digestive system diseases, and 13% mental disorders.  ^§§^Anatomical Therapeutic Chemical Classification System.  Abbreviations. BMI: body mass index; FEV_1_pp: forced expiratory volume in one-second percentage predicted; FVCpp: forced vital capacity percentage predicted; PA: physical activity. | | | |
