# Supplementary material for: Physical activity coaching programme for people with Long COVID: a pilot randomised clinical trial
Source: Sci Rep. 2026 Mar 24;16:14820. doi: 10.1038/s41598-026-44806-9 (PMC13168267; doi:10.1038/s41598-026-44806-9)
Supplement: Supplementary file 3 — Supplementary Information 3. [file 41598_2026_44806_MOESM3_ESM.docx]

### **e-TABLE 2.** **Participants’ adverse events at 3-month and 6-month assessments from the physical activity coaching programme for people with Long COVID**

|  | Per-protocol | | | | Intention-to-treat | | | | |
| --- | --- | --- | --- | --- | --- | --- | --- | --- | --- |
|  | **All** | **Usual care** | **PA coaching** | **Sig.** | **All** | **Usual care** | **PA coaching** | **Sig.** |  |
| **Subjects n** | **40** | **21** | **19** |  | **50** | **25** | **25** |  |  |
| **3-month assessment** | | | | | | | | |  |
| Reinfections n(%) | 6(15) | 3(14) | 3(16) | p=1.00 | 6(12) | 3(12) | 3(12) | p=1.00 |  |
| Exacerbations n(%) | 25(63) | 12(57) | 13(68) | p=0.53 | 29(58) | 12(48) | 17(68) | p=0.34 |  |
| Exacerbations median(Q1;Q3) | 1(0;3) | 1(0;5) | 2(0;3) | p=0.71 | 1(0;3) | 1(0;5) | 1(0;3) | p=0.59 |  |
| Emergency service use n(%) | 13(33) | 9(43) | 4(21) | p=0.19 | 14(28) | 9(36) | 5(20) | p=0.20 |  |
| Emergency service use median(Q1;Q3) | 0(0;1) | 0(0;1) | 0(0;0) | p=0.19 | 0(0;1) | 0(0;1) | 0(0;0) | p=0.09 |  |
| Hospitalisations n(%) | 1(3) | 1(5) | 0(0) | p=1.00 | 1(2) | 1(4) | 0(0) | p=0.48 |  |
| Hospitalisations median(Q1;Q3) | 0(0;0) | 0(0;0) | 0(0;0) | p=0.81 | 0(0;0) | 0(0;0) | 0(0;0) | p=0.30 |  |
| **6-month assessment** | | | | | | | | |  |
| Reinfections n(%) | 1(3) | 1(5) | 0(0) | p=1.00 | 1(2) | 1(4) | 0(0) | p=0.48 |  |
| Exacerbations n(%) | 24(60) | 12(57) | 12(63) | p=0.76 | 26(52) | 12(48) | 14(56) | p=1.00 |  |
| Exacerbations median(Q1;Q3) | 1(0;2) | 1(0;2) | 1(0;2) | p=0.87 | 1(0;2) | 1(0;2) | 1(0;2) | p=0.96 |  |
| Emergency service use n(%) | 12(30) | 6(29) | 6(32) | p=1.00 | 12(24) | 6(24) | 6(24) | p=1.00 |  |
| Emergency service use median(Q1;Q3) | 0(0;1) | 0(0;1) | 0(0;1) | p=0.94 | 0(0;1) | 0(0;1) | 0(0;1) | p=0.65 |  |
| Hospitalisations n(%) | 1(3) | 1(5) | 0(0) | p=1.00 | 1(2) | 1(4) | 0(0) | p=0.48 |  |
| Hospitalisations median(Q1;Q3) | 0(0;0) | 0(0;0) | 0(0;0) | p=0.81 | 0(0;0) | 0(0;0) | 0(0;0) | p=0.30 |  |
| Abbreviations: PA: physical activity. | | | | | | | | | |
