# Supplementary material for: Physical activity coaching programme for people with Long COVID: a pilot randomised clinical trial
Source: Sci Rep. 2026 Mar 24;16:14820. doi: 10.1038/s41598-026-44806-9 (PMC13168267; doi:10.1038/s41598-026-44806-9)
Supplement: Supplementary file 4 — Supplementary Information 4. [file 41598_2026_44806_MOESM4_ESM.docx]

**e-TABLE 3. Preliminary efficacy and effectiveness of physical activity coaching vs. usual care in people with Long COVID, adjusted data**

|  |  | Per-protocol | | | | | | | | Intention-to-treat | | | | | |
| --- | --- | --- | --- | --- | --- | --- | --- | --- | --- | --- | --- | --- | --- | --- | --- |
|  | **Time point** | **Usual care** | | **PA coaching** | **MD[95%CI] or RD[95%CI]** | | | **Sig. group*time** | | **Usual care** | **PA coaching** | | **MD[95%CI] or RD[95%CI]** | **Sig. group*time** | |
| Subjects n |  | **21** | | **19** |  | | |  | | **25** | **25** | |  |  | |
| Secondary outcomes^#^ | | |  | | |  |  | |  | | |  |  |  |  |
| LPA, min·day^-1^ | Baseline  3 months  6 months | 134±16  117±14  105±15 | | 137±17  156±15  155±16 | -3[-53;48]  -39[-85;7]  -50[-97;-3]* | | | p=0.06 | | 126±14  112±14  102±14 | 128±14  142±13  142±14 | | -2[-46;41]  -30[-72;12]  -41[-84;3] | p=0.17 | |
| MVPA, min·day^-1^ | Baseline  3 months  6 months | 3±1  4±2  4±2 | | 4±1  8±2†  10±2† | -2[-6;3]  -4[-8;1]  -6[-12;1] | | | p=0.11 | | 3±1  5±2  5±2 | 4±1  8±2†  9±2† | | -1[-4;3]  -3[-8†2]  -5[-11;1] | p=0.21 | |
| Steps·day^-1^ | Baseline  3 months  6 months | 5944±847  5434±816  5251±856 | | 6605±905  9324±876†  9141±914† | -660[-3416;2095]  -3890[-6583;-1197]*  -3890[-6665;-1115]* | | | p<0.01* | | 5794±706  5301±745  5160±810 | 6267±709  8476±734†  8325±791† | | -473[-2661;1716]  -3176[-5437;-914]*  -3165[-5562;-767]* | p<0.01* | |
| Steps·day^-1^≤5000 n(%) | Baseline  3 months  6 months | 10(49)  12(63)  13(68) | | 6(28)  2(11)  3(17) | 22[-9;53]  52[26;79]*  52[24;79]* | | | p=0.16 | | 13(52)  12(64)  13(69) | 9(36)  4(23)  4(26) | | 16[-12;45]  41[14;68]*  43[16;70]* | p=0.52 | |
| Sedentary time, min·day^-1^ | Baseline  3 months  6 months | 661±26  670±28  676±26 | | 684±27  631±29†  618±28† | -23[-106;60]  39[-48;126]  58[-26;142] | | | p=0.02 | | 664±23  673±26  677±25 | 677±23  633±26†  620±25† | | -13[-85;58]  40[-37;117]  57[-18;132] | p=0.03 | |
| 6MWD, m | Baseline  3 months  6 months | 457±29  440±29  441±29 | | 476±31  521±31†  535±31† | -19[-115;77]  -81[-177;15]  -94[-189;2] | | | p<0.01* | | 459±26  444±26  446±26 | 484±26  527±26†  541±26† | | -25[-105;56]  -84[-164;-3]*  -95[-176;-15]* | p<0.01* | |
| 6MWD≤70pp n(%) | Baseline  3 months  6 months | 9(42)  13(64)  13(64) | | 10(54)  3(13)†  2(2)† | -12[-44;20]  51[25;78]*  62[38;86]* | | | p=0.05 | | 10(40)  13(62)†  13(62)† | 11(44)  5(17)†  4(12)† | | -4[-33;24]  46[19;72]*  51[22;79]* | p=0.12 | |
| 1minSTS, reps | Baseline  3 months  6 months | 21±3  23±3  23±3 | | 27±3  30±3†  31±3† | -5[-14;4]  -7[-16;2]  -8[-17;1] | | | p=0.45 | | 22±2  24±2  23±2 | 25±2  28±2†  29±2† | | -3[-10;4]  -5[-12;3]  -6[-13;1] | p=0.36 | |
| 1minSTS≤70pp n(%) | Baseline  3 months  6 months | 16(76)  13(60)  14(70) | | 9(47)  8(43)  6(26) | 29[-2;59]  18[-15;51]  44[15;73]* | | | p=0.04 | | 18(72)  13(57)  14(66) | 14(56)  11(51)  9(41) | | 15[-12;42]  6[-25;36]  25[-3;53] | p=0.10 | |
| QMVC, kgf | Baseline  3 months  6 months | 21±2  20±2  19±2 | | 20±2  22±2  22±2 | 1[-6;7]  -2[-9;4]  -3[-9;4] | | | p=0.13 | | 23±2  22±2  21±2 | 19±2  21±2  21±2 | | 3[-2;9]  0[-5;6]  0[-5;5] | p=0.07 | |
| QMVC≤70pp n(%) | Baseline  3 months  6 months | 4(19)  4(18)  5(23) | | 2(8)  3(6)  2(5) | 11[-9;32]  12[-8;33]  18[4;40] | | | p=0.17 | | 4(16)  4(15)  5(20) | 3(8)  3(14)  2(8) | | 7[-11;25]  1[-21;24]  12[-9;34] | p=0.05 | |
| Handgrip, kgf | Baseline  3 months  6 months | 26±3  26±2  26±2 | | 20±3  23±3  23±3† | 6[-2;15]  4[-4;12]  3[-5;11] | | | p=0.05 | | 27±2  27±2  27±2 | 21±2  24±2†  25±2† | | 5[-1;12]  3[-3;9]  2[-5;9] | p=0.06 | |
| Handgrip ≤70pp n(%) | Baseline  3 months  6 months | 10(49)  12(59)  13(67) | | 11(58)  7(33)  6(29)† | -9[-42;24]  26[-7;60]  38[5;71]* | | | p=0.60 | | 11(44)  12(57)  13(65) | 13(52)  7(27)†  6(24)† | | -8[-38;21]  30[-1;61]  41[10;71]* | p=0.52 | |
| PImax, cmH2O | Baseline  3 months  6 months | 65±6  61±5  58±5 | | 68±6  71±6  73±5 | -3[-21;16]  -11[-28;7]  -15[-31;1] | | | p=0.19 | | 68±5  62±5  60±4 | 66±5  68±5  70±4 | | 2[-13;17]  -6[-20;9]  -10[-24;4]g | p=0.10 | |
| PImax≤70pp n(%) | Baseline  3 months  6 months | 15(70)  17(85)  19(100)† | | 12(64)  10(49)  10(50) | 6[-26;37]  36[0;71]*  49[21;77]* | | | p<0.01* | | 18(70)  17(84)  19(100)† | 17(69)  14(64)  14(63) | | 2[-25;28]  20[-10;49]  36[14;58]* | p=0.02* | |
| PEmax, cmH2O | Baseline  3 months  6 months | 74±8  80±6  80±6 | | 85±8  102±7†  107±7† | -11[-35;12]  -22[-43;-1]*  -27[-48;-6]* | | | p=0.16 | | 80±7  84±6  84±6 | 81±7  98±6†  102±6† | | -1[-21;19]  -14[-32;4]  -18[-37;0]* | p=0.07 | |
| PEmax≤70pp n(%) | Baseline  3 months  6 months | 19(91)  21(100)  21(100) | | 17(89)  13(73)  11(57)† | 2[-17;21]  27[0;55]*  43[17;69]* | | | p<0.01* | | 22(88)  21(100)  21(100) | 23(92)  17(79)  15(66)† | | -4[-21;14]  21[2;41]*  34[13;56]* | p<0.01* | |
| Dyspnoea mMRC | Baseline  3 months  6 months | -  2(2;2)  2(1;2) | | -  1(1;1)†  1(1;1)† | 1[-1;1]  1[0;1]*  1[0;1]* | | | p<0.01* | | -  2(2;2)  2(1;2) | -  1(1;1)†  1(1;1)† | | 2[0;0]  1[0;1]*  1[0;1]* | p<0.01* | |
| mMRC≥2 n(%) | Baseline  3 months  6 months | 17(100)  15(92)  15(92) | | 9(34)  1(1)  0(0) | 66[34;98]*  90[74;100]*  92[75;100]* | | | p<0.01* | | 20(80)  15(60)  15(60) | 11(44)  1(4)†  0(0)† | | -  -  - | p<0.01* | |
| Fatigue FACIT-FS | Baseline  3 months  6 months | 22(19;24)  22(15;26)  21(16;28) | | 16(8;21)  29(22;32)†  35(32;39)† | 5[1;10]*  -5[-9;0]*  -10[-14;-6]* | | | p<0.01* | | 19(16;24)  22(16;26)  21(15;25) | 17(9;22)  28(21;32)†  35(32;40)† | | 4[-1;9]  -6[-10;-1]*  -10[-15;-5]* | p<0.01* | |
| FACIT-FS≤43 n(%) | Baseline  3 months  6 months | 21(100)  21(100)  21(100) | | 19(100)  18(95)†  13(68)† | -  -  - | | | p<0.01* | | 24(96)  21(84)  21(84) | 25(100)  22(88)†  16(64)† | | -  -  - | p<0.01* | |
| PEM DSQ n(%) | Baseline  3 months  6 months | 19(91)  18(92)  20(96) | | 18(100)  13(68)†  12(63)† | -9[-23;5]  24[-1;49]  33[10;57]* | | | p=0.06 | | 23(92)  18(92)  20(96) | 24(100)  17(74) †  15(65)† | | -8[-20;4]  18[-4;40]  31[10;52]* | p=0.03 | |
| ME/CFS DSQ n(%) | Baseline  3 months  6 months | 11(52)  15(71)  15(71) | | 14(74)  4(21)†  0(0)† | -  -  - | | | p<0.01* | | 13(52)  15(76)  15(76) | 19(76)  5(21)†  1(1)† | | -24[-50;2]  55[26;84]*  75[50;99]* | p=0.10 | |
| Anxiety HADS-A | Baseline  3 months  6 months | 8(6;10)  6(4;10)  6(5;9) | | 10(7;14)  11(6;16)  10(6;15) | -3[-7;0]  -2[-6;1]  -3[-6;1] | | | p=0.54 | | 8(6;10)  7(4;10)  7(4;10) | 11(7;12)  10(6;15)  11(6;15) | | -3[-6;0]  -2[-5;1]  -2[-6;1] | p=0.52 | |
| HADS-A≥8 n(%) | Baseline  3 months  6 months | 9(43)  9(43)  10(48) | | 14(76)  12(63)  12(63) | -33[-64;1]  -20[-52;12]  -15[-47;17] | | | p=0.60 | | 11(44)  9(43)  10(48) | 18(75)  14(61)  14(61) | | -31[-58;-4]*  -18[-48;13]  -12[-42;18] | p=0.64 | |
| Depression HADS-D | Baseline  3 months  6 months | -  8(5;10)  9(5;10) | | -  9(7;14)  8(6;13) | -1[-5;2]  -2[-5;2]  -2[-5;1] | | | p=0.30 | | -  8(6;10)  8(5;9)† | -  10(8;13)  9(6;13) | | -1[-4;2]  -1[-4;2]  -1[-4;2] | p=0.24 | |
| HADS-D≥8 n(%) | Baseline  3 months  6 months | 14(68)  12(60)  11(56) | | 11(58)  12(63)  11(58) | 11[-21;43]  -4[-37;29]  -2[-37;34] | | | p=0.40 | | 17(69)  12(59)  11(55) | 16(65)  16(69)  14(61) | | 4[-23;31]  -10[-40;20]  -6[-38;27] | p=0.34 | |
| EQ-5D-5L | Baseline  3 months  6 months | 50(41;60)  44(21;49)  42(31;53) | | 37(33;51)  56(50;64)†  60(59;72)† | 8[-2;18]  -10[-20;-1]*  -15[-24;-7]* | | | p<0.01* | | -  43(34;50)  42(32;52) | 56(54;66)†  61(59;70)† | | 3[-8;13]  -15[-24;-6]*  -19[-27;-11]* | p<0.01* | |
| EQ-5D-5L≤threshold n(%) | Baseline  3 months  6 months | 19(91)  20(95)  21(100) | | 19(100)  17(90)  15(79) | -  -  - | | | p<0.01* | | 22(89)  20(94)  21(100) | 25(100)  20(90)  18(84) | | -11[-26,4]  4[-13;21]  16[-3;34] | p=0.04 | |
| Data are reported as mean±SE or median(Q1;Q3), unless otherwise stated.  Continuous data and proportions are adjusted for time from acute COVID-19, baseline lung function (FEV_1_pp and FVCpp values), and dyspnoea severity (mMRC score).  Risk difference represents the absolute difference in proportions between groups.  *Statistically significant p-value <0.017 between groups, †Statistically significant p-value <0.05 within each group  Abbreviations. 1minSTS: 1-min sit-to-stand test; 6MWD: 6-min walking distance; DSQ: DePaul symptom questionnaire; EQ-5D-5L: European quality of life - 5 dimensions - 5 levels; FACIT-FS: functional assessment of chronic illness therapy - fatigue; HADS: hospital anxiety and depression scale; LPA: time spent in light physical activity; MD: mean difference; ME/CFS: myalgic encephalomyelitis/chronic fatigue syndrome; mMRC: modified Medical Research Council; MVPA: time spent in moderate-to-vigorous physical activity; PImax: maximal inspiratory pressure; PEM: post-exertional malaise; PEmax: maximal expiratory pressure; QMVC: quadriceps muscle voluntary contraction; RD: risk difference. | | | | | | | | | | | | | | |  |
